# Using self-supervised feature learning to improve the use of pulse oximeter signals to predict paediatric hospitalization

**DOI:** 10.12688/wellcomeopenres.17148.2

**Published:** 2023-02-01

**Authors:** Paul Mwaniki, Timothy Kamanu, Samuel Akech, Dustin Dunsmuir, J. Mark Ansermino, M.J.C Eijkemans

**Affiliations:** 1 Kenya Medical Research Institute/Wellcome Trust Research Programme, Nairobi, Kenya; 2 School of Mathematics, University of Nairobi, Nairobi, Kenya; 3 Digital Health Innovation Lab, BC Children’s Hospital Research Institute, Vancouver, British Columbia, Canada; 4 Department of Anesthesiology, Pharmacology & Therapeutics, University of British Columbia, Vancouver, British Columbia, Canada; 5 Julius Center for Health Sciences and Primary Care, Department of Data Science and Biostatistics, University Medical Center Utrecht, Utrecht University, Utrecht, The Netherlands

**Keywords:** Signal processing, Self-supervised learning, Photoplethysmography, Deep learning, Hospitalization

## Abstract

**Background**: The success of many machine learning applications depends on knowledge about the relationship between the input data and the task of interest (output), hindering the application of machine learning to novel tasks. End-to-end deep learning, which does not require intermediate feature engineering, has been recommended to overcome this challenge but end-to-end deep learning models require large labelled training data sets often unavailable in many medical applications. In this study, we trained self-supervised learning (SSL) models for automatic feature extraction from raw photoplethysmography (PPG) obtained using a pulse oximeter, with the aim of predicting paediatric hospitalization.

**Methods**: We compared logistic regression models fitted using features extracted using SSL with models trained using both clinical and SSL features. In addition, we compared end-to-end deep learning models initialized randomly or using weights from the SSL models. We also compared the performance of SSL models trained on labelled data alone (n=1,031) with SSL trained using both labelled and unlabelled signals (n=7,578).

**Results**: Logistic regression models were more predictive of hospitalization when trained on features extracted using labelled PPG signals only compared to SSL models trained on both labelled and unlabelled signals (AUC 0.83 vs 0.80). However, features extracted using SSL model trained on both labelled and unlabelled PPG signals were more predictive of hospitalization when concatenated with clinical features (AUC 0.89 vs 0.87). The end-to-end deep learning model had an AUC of 0.80 when initialized using the SSL model trained on all PPG signals, 0.77 when initialized using SSL trained on labelled data only, and 0.73 when initialized randomly.

**Conclusions**: This study shows that SSL can extract features from PPG signals that are predictive of hospitalization or initialize end-to-end deep learning models. Furthermore, SSL can leverage larger unlabelled data sets to improve performance of models fitted using small labelled data sets.

## Introduction

Pulse oximeters are used in routine clinical practice to detect hypoxia (low blood oxygen) and measure heart rate ( WHO, 2016). Photoplethysmography (PPG) is the underlying mechanism employed by pulse oximeters. The resulting PPG waveform contains additional information on the respiratory and cardiovascular systems that can help diagnose various medical conditions or pathophysiological states.

Analysis of bio-signals such as PPG typically involves signal pre-processing, feature extraction, and classification or regression using the extracted features. Filters are applied to the signal during the pre-processing stage to eliminate noise such as motion artefacts before hand-crafted features required for regression or classification are extracted. Such features are extracted from signals in the original time-domain or after frequency decomposition using Fourier or wavelet transform ( Cvetkovic *et al.*, 2008). Hand-crafted features require domain knowledge on the relationship between the waveform and the task of interest, limiting the application of bio-signals to novel tasks.

Deep learning models can bypass signal pre-processing and feature extraction required for classification and regression tasks, speeding up model development in tasks where the relationship between the raw signal and outcome of interest is not well understood in a process referred to as end-to-end deep learning. End-to-end deep learning models have been applied successfully to computer vision (CV), natural language processing (NLP), and raw audio signals ( Devlin *et al.*, 2019; He *et al.*, 2015; Schmidhuber, 2015). Automatic feature extraction and classification using end-to-end deep learning can be used to analyse PPG signals, but the models are likely to overfit when trained on small data sets ( Bian *et al.*, 2020; Panwar *et al.*, 2020).

Pre-trained models for either feature extraction or model initialization have been shown to improve models trained using small labelled data sets in CV and NLP tasks, but such models are generally unavailable for other tasks ( del Rio *et al.*, 2018; Oquab *et al.*, 2014). In this study, we used self-supervised learning (SSL) methods that do not require the availability of pre-trained models. SSL uses a pretext task that learns useful representation (features) from the inputs and a downstream task that solves the problem of interest using the extracted features. Pretext tasks are either generative or discriminative tasks; generative models such as auto-encoders aim to reconstruct the input or generate data from the same distribution as inputs, whilst discriminative approaches such as contrastive learning derive labels from the inputs. An example of contrastive learning in CV is instance classification, where each image in the training data set is treated as a class, and multiple views of each image are obtained through stochastic image augmentation ( Chen *et al.*, 2020; He *et al.*, 2020). An encoder model is then trained so that the distances between the embedding of positive pairs (views from the same image) are closer compared to those of negative pairs, which are views from different images. Self-supervised learning using contrastive methods has been used for extracting features from electroencephalogram (EEG) signals, and could be useful for PPG signals too ( Banville *et al.*, 2019; Banville *et al.*, 2020). Therefore, we used contrastive learning where randomly sampled segments from the same patient were treated as positive pairs, and those from different patients as negative pairs.

This study explores the utility of SSL learning in extracting features from raw PPG signals and initializing end-to-end deep learning models to predict hospitalization given raw PPG signals. We use features extracted using contrastive learning to predict hospitalization using logistic regression. We also relate extracted features to known physiological parameters such as respiratory rate, heart rate and oxygen saturation (SpO _2_), which are associated with severity of illness using linear regression. We compare end-to-end deep learning models for predicting hospitalization initialized randomly with those initialized using weights of the SSL model. Finally, we assess the utility of an unlabelled PPG signal data set sourced from a different study in improving SSL models. That is, we compared the prediction performance of features extracted using an SSL model trained on labelled data alone with an SSL model trained using both labelled and unlabelled data.

## Methods

### Study setting and data

***Labelled PPG signals.*** The study was conducted at the paediatric outpatient department of Mbagathi county referral hospital in Nairobi, Kenya, between January and June 2018. The study nurse recruited eligible children aged 2–60 months presenting with medical illnesses before they were reviewed by the attending clinician. The study nurse collected raw PPG signals using a pulse oximeter connected to an android tablet. PPG signals were collected for up to 60 seconds for each patient, and respiratory rate was obtained using an android application that estimated respiratory rates by tapping the tablet's screen with every respiratory cycle ( Karlen *et al.*, 2014). The study nurse recorded study participants’ demographic information together with clinical signs and symptoms. The facility physicians, who were not part of the study and without access to the study data, decided on whether to admit any patient. The study nurse followed up on patients who were not admitted via telephone calls approximately two weeks after the hospital visit to determine if the patient recovered from the ailment without required admission later or in a different facility. More details about study procedures are described elsewhere ( Mawji *et al.*, 2021).

The study collected PPG signals from 1,031 children, of whom 125 (12.2%) were hospitalized. The median age of recruited children was 16 months with an IQR of 9 – 30 months. The PPG signals had median signal quality index (SQI) of 80.5% (IQR 57.25% - 93.0%). Data from 20% of the children were set aside for final model performance evaluation, while the rest was used for model training and validation.

This study was approved by the Scientific and Ethics Review Unit (SERU) of the Kenya Medical Research Institute (certificate number, SERU/3407). The study nurse obtained written informed consent from the caregiver of recruited children before data collection except in emergency cases where consent was obtained after the child was stable to avoid delaying emergency care.

***Unlabelled PPG signals.*** We obtained additional unlabelled PPG signals from the Smart Discharges (SD) study following an open access request to the Pediatric Sepsis CoLab to evaluate the utility of additional unlabelled data in improving the performance of self-supervised learning models ( Wiens *et al*., 2021a; Wiens *et al*., 2021b). We compared self-supervised models fitted using labelled data alone with models fitted using both labelled and unlabelled PPG signals. The SD study recruited children below 60 months old, hospitalized in six public hospitals in Uganda with suspected or confirmed infection. The PPG signals were treated as unlabelled despite being collected from hospitalized children because they were not collected at triage or soon after the patient presented at the hospital. The study staff recorded two 60 seconds PPG signals at both admission and discharge. There were 2707 children aged below six months and 3840 aged between 6 and 60 months.

### PPG signal acquisition and pre-processing

The pulse oximeters used for PPG signal acquisition for both Kenya and Uganda studies were developed by Lionsgate Technologies Medical (Vancouver, Canada) and made use of photoplethysmographic pulses in the red and infrared wavelengths. The tablet application saved PPG signals that had been downsampled to 80Hz for later analysis.

 We split the PPG signals into segments of 10 seconds with two seconds overlap between consecutive segments and normalized each channel using mean and standard deviation calculated from the Mbagathi hospital (labelled) data set.

### Feature extraction using contrastive learning

We fitted an encoder to extract features from raw PPG signals using Pytorch 1.7 ( Paszke *et al.*, 2019). The architecture for the encoder was similar to ResNet50 with several modifications ( Zagoruyko & Komodakis, 2017). We used one-dimensional convolution layers because PPG signals are one dimensional. The first convolutional layer had two channels instead of three because PPG signals have two channels (red and infrared) instead of the red, blue, and green channels in colour images. Max-pooling was applied after every convolutional layer group to increase the network's receptive field and reduce computation cost. The fully connected layer of ResNet50 was replaced with two fully connected layers with 32 units each. Similar to ( Chen *et al.*, 2020), the last fully connected layer was discarded after training.

Two contrastive learning models were trained: one model was trained using PPG signals from Mbagathi hospital only (labelled data), and the other was trained using PPG signals from both Mbagathi hospital and unlabelled PPG signals from the SD study. The two contrastive models were used to extract features for classification using logistic regression and initialize the weights of the end-to-end deep learning models.

### Pretext task

The encoder was trained so that embeddings of two segments from the same patient, referred here as positive pairs, were closer than those of two segments from different patients (negative pairs). The unlabelled data from the SD study had multiple PPG recordings for each patient: two admission and two discharge recordings. We treated the admission and discharge recordings as coming from different patients because a substantial amount of time may have passed between the recordings (hospitalization length of stay). A mini-batch would consist of N pairs of segments during training, with each pair sampled from segments from the same patient. Similar to ( Chen *et al.*, 2020; He *et al.*, 2020), negative pairs for a given patient were obtained by taking the 2(N-1) segments in the mini-batch that did not belong to that patient. We also tried a different pretext task that classified whether two PPG segments are obtained from multiple augmentations of the same PPG segment. This pretext task was not able to learn useful representations of the PPG signals and was therefore dropped.

Noise contrastive estimation (NCE) loss was used to train the encoder ( Gutmann & Hyvärinen, 2010). Dot product was used to measure the distance between two embeddings. The contrastive loss for segment j in a mini-batch of N pairs of segments is shown in Equation 1.


$$l_{j}=-\log\frac{\exp(q.k_{+}\text{/}\tau )}{\sum_{i=0}^{2(N-1)}\exp(q.k_{i}\text{/}\tau )}\;(\text{Equation}\;1)$$

where *q* is a query embedding, *k*_+_ is the embedding from the positive pair, *k*_i_ are the embeddings of 2(N-1) negative and one positive segments, and τ is a temperature hyper-parameter.

Data augmentation was applied to PPG signals in the training data set as part of the model training pipeline. Data augmentation was done by adding Gaussian noise to the signals and window slicing and permutation ( Um *et al.*, 2017). Gaussian noise with standard deviation ranging between 1e-5 and 1e-2 was added to a proportion of training signals. Window slicing and permutation was done by varying the number of windows and the proportion of augmented signals.

Hyper-parameter tuning was done using Asynchronous Successive Halving Algorithm (ASHA) using the ray-tune library in Python ( Li *et al.*, 2018; Liaw *et al.*, 2018). The tuned hyper-parameters included regularization parameters (dropout and weight decay), data augmentation (proportion of signals with augmentation and the number of slices), batch size, and temperature parameter of NCE loss.

Extracted features were evaluated qualitatively using t-distributed stochastic network embeddings (t-SNE). Three scatter plots of the first, second and third components of t-SNE were shaded using values of heart rate, respiratory rate and SpO _2_. Features for each patient were obtained by averaging embedding of all segments belonging to that patient.

### Classification and regression using features extracted using contrastive learning

Features extracted using SSL were used to predict hospitalization using logistic regression. We also evaluated the utility of extracted features in improving logistic regression models trained using eight clinical features identified from previous analysis ( Mawji *et al.*, 2021). The eight features include weight, restlessness, mid-upper arm circumference (MUAC), ability to drink/breastfeed, SpO _2_, temperature, difficulty breathing, and heart rate. We concatenated the 32 features extracted using contrastive learning and the eight clinical features. We also assessed the quality of extracted features by using linear regression to predict physiological parameter know to be present in PPG signals (heart rate, respiratory rate and SpO _2_). We fitted Bayesian logistic and linear regression models using the Pyro library in python ( Bingham *et al.*, 2018). Horseshoe priors were used to induce sparsity in the coefficients of logistic and linear regression to prevent over-fitting ( Carvalho *et al.*, 2009). Model performance was assessed using the area under the curve (AUC) for classification models, and root mean square error (RMSE) and coefficient of determination (R2) for regression models.

### End-to-end classification using deep learning

The end-to-end deep learning models for predicting hospitalization were identical to the encoder used for contrastive learning, except that the last fully connected layer had a single output. We compared end-to-end deep learning models initialized randomly with those initialized using weights learned by contrastive learning models. The models were trained on soft labels (label smoothing) recommended for noisy targets, and the minority class was oversampled to address class imbalance ( Müller *et al.*, 2019; Szegedy *et al.*, 2015). We tuned hyper-parameters for regularization (dropout, weight decay, data augmentation), batch size, learning rate, and label smoothing parameter using the ASHA algorithm. Python code for the analysis is available on Github ( https://github.com/pmwaniki/ppg-analysis/releases/tag/v1.0.1).

## Results

**Figure 1.**
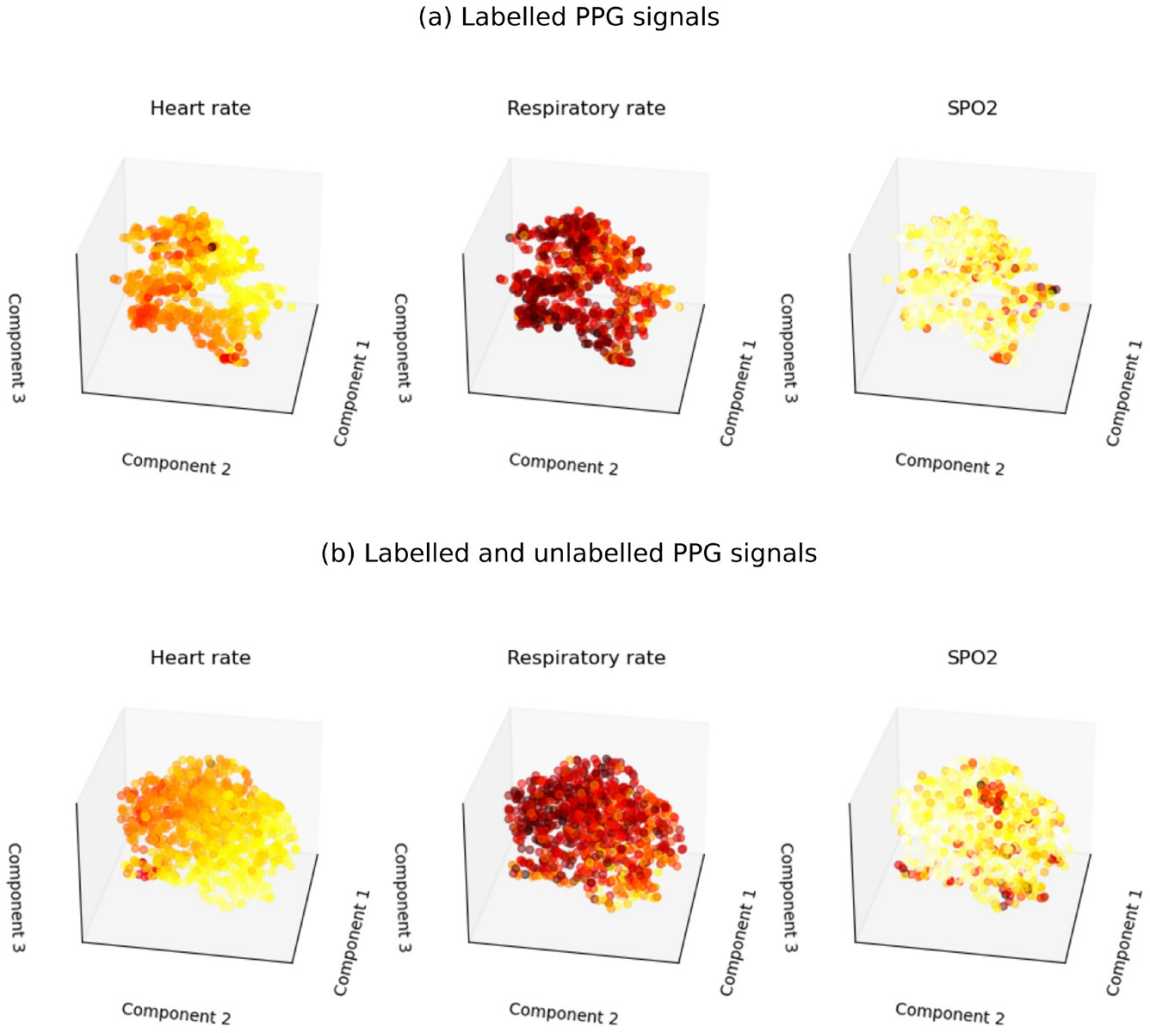
t-SNE visualizations of features extracted using SSL shaded by heart rate, respiratory rate and SpO _2_. ( **a**) Features are extracted using the SSL model fitted using labelled PPG signals only. ( **b**) Features are extracted using the SSL model fitted using both labelled and unlabelled PPG signals.

**Figure 2.**
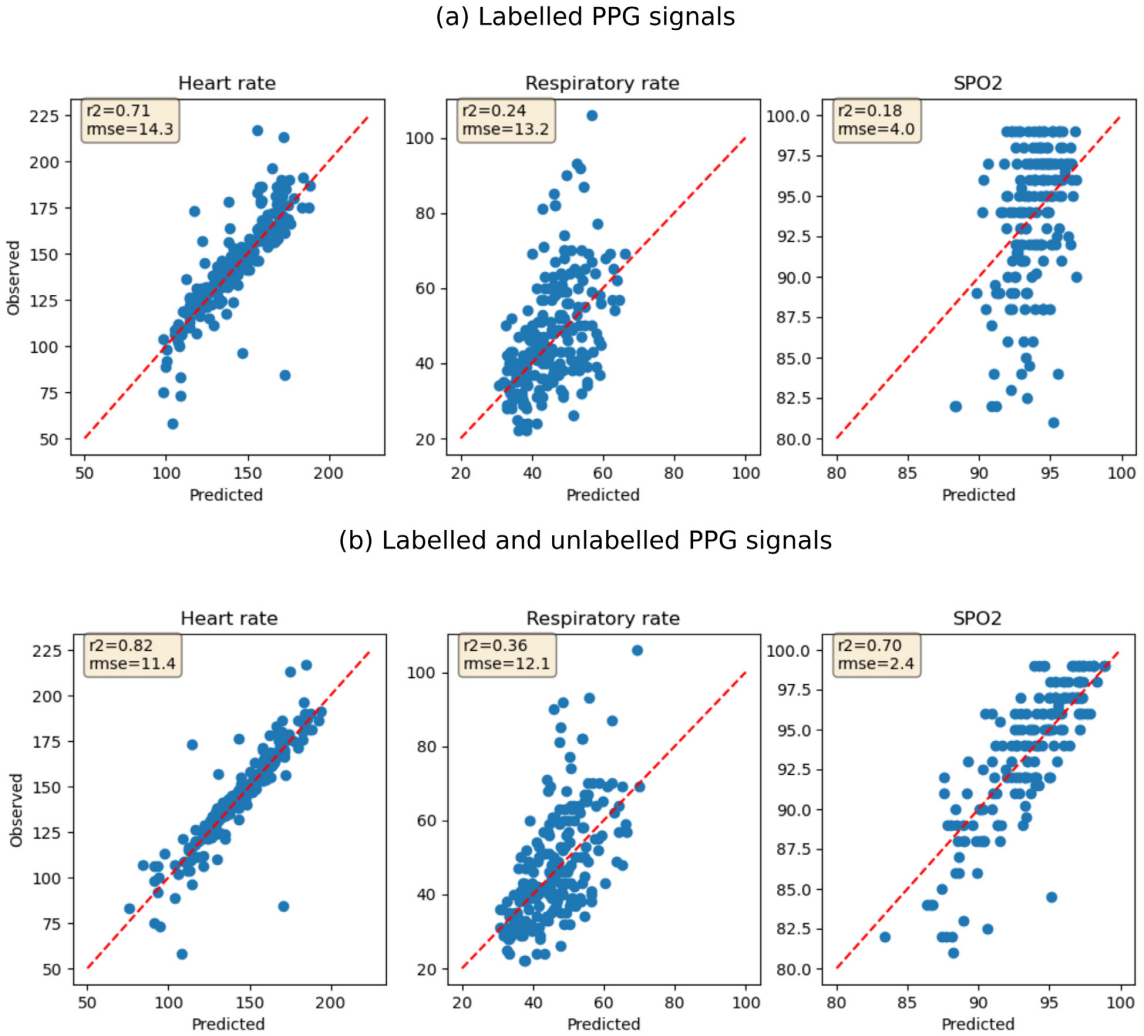
Performance of linear regression models predicting heart rate, respiratory rate and SpO _2_ using features extracted using SSL. ( **a**) Features are extracted using SSL model fitted using labelled PPG signals only. ( **b**) Features are extracted using SSL model fitted using both labelled and unlabelled PPG signals. The red dotted line shows test data points where the predicted value equals the observed value (perfect prediction).

The contrastive learning model trained on both labelled and unlabelled PPG signals had higher accuracy in classifying whether two PPG segments were obtained from the same patient than the model trained on labelled data only (validation accuracy, 91% vs 73%). Qualitative evaluation of extracted features using t-SNE showed that the features were correlated with heart rate, respiratory rate, and SpO _2_. Data point with similar values of heart rate, respiratory rate, or SpO _2_ were clustered together on the t-SNE plot ( Figure 1). Quantitative evaluation using linear regression showed that features extracted using the SSL model trained on both labelled and unlabelled data were more predictive of physiological parameters compared to features extracted using labelled data only: heart rate (R2 0.82 vs 0.71), respiratory rate (R2 0.36 vs 0.24) and SpO2 (R2 0.70 vs 0.18). However, the regression models under-estimated respiratory rate measurements above 70 breaths per minute and overestimated SpO _2_ measurements below 87 ( Figure 2). Logistic regression models for predicting hospitalization using the extracted features had AUC of 0.83 (sensitivity 0.88 & specificity 0.50) for SSL model trained on labelled data only and 0.80 (sensitivity 0.73 & specificity 0.64) for SSL model trained on both labelled and unlabelled data. Logistic regression models trained using the eight clinical features had an AUC of 0.86 (sensitivity 0.81 & specificity 0.77). Concatenating the eight clinical features and features extracted using contrastive learning improved AUC to 0.87 (sensitivity 0.81 & specificity 0.79) for SSL models trained on labelled data only and AUC to 0.89 (sensitivity 0.85 & specificity 0.75) for SSL models trained using both labelled and unlabelled PPG signals. Logistic regression models trained on heart rate and SpO _2_ only had AUC of 0.72 (sensitivity 0.69 & specificity 0.65). Logistic regression models trained on features extracted using SSL, SpO _2_, and heart rate had the same AUCs as models trained on SSL features only. End-to-end deep learning models for predicting hospitalization had an AUC of 0.73 (sensitivity 0.72 & specificity 0.58) when initialized randomly. The AUC of end-to-end models rose to 0.77 (sensitivity 0.81 & specificity 0.56) when initialized using the SSL model trained on labelled data only, and 0.80 (sensitivity 0.85 & specificity 0.67) for model initialized using the SSL model trained on both labelled and unlabelled data ( Table 1).

**Table 1.** Performance of classification models for predicting hospitalization. SSL models are used to extract features for classification using logistic regression models and initializing end-to-end deep learning models. Comparison are made between deep learning models with different initialization schemes with logistic regression models fitted using various combinations of clinical features and features extracted using SSL.

Model	Initialization/Features	Precision	Sensitivity	Specificity	AUC
**Deep learning**	**Random**	0.20	0.73	0.58	0.73
	**SSL(Labelled & Unlabelled)**	0.27	0.85	0.67	0.80
	**SSL(Labelled)**	0.21	0.81	0.56	0.77
**Logistic regression**	**Clinical**	0.34	0.81	0.77	0.86
	**Clinical & SSL(Labelled & Unlabelled)**	0.33	0.85	0.75	0.89
	**Clinical & SSL(Labelled)**	0.36	0.81	0.79	0.87
	**SPO2 & heart rate**	0.22	0.69	0.65	0.72
	**SPO2, heart rate & SSL(Labelled & Unlabelled)**	0.24	0.77	0.64	0.80
	**SPO2, heart rate & SSL(Labelled)**	0.20	0.88	0.50	0.83
	**SSL(Labelled & Unlabelled)**	0.23	0.73	0.64	0.80
	**SSL(Labelled)**	0.20	0.88	0.50	0.83

## Discussion

### Key findings

This study sought to evaluate the utility of SSL in extracting features from raw PPG signals and initializing end-to-end deep learning models with the aim of predicting hospitalization. Moreover, a comparison is made between SSL models trained using labelled data only with models trained with additional unlabelled data from a different setting. Self-supervised models performed better when trained on labelled and unlabelled data despite the unlabelled data set originating from a different setting. Features extracted using SSL model trained using labelled and unlabelled data were more predictive of heart rate, respiratory rate, and SpO _2_ than features extracted using the model trained on labelled data only. While features extracted using SSL models trained on labelled data only were better at predicting hospitalization than features extracted using SSL models trained on both labelled and unlabelled data, features extracted using both labelled and unlabelled data improved models trained on clinical features more. Weights from SSL models trained on both labelled and unlabelled data were better at initializing end-to-end models. Weights from the self-supervised model trained on labelled data alone were better at initializing end-to-end models than random initialization, suggesting that self-supervised models may still be useful when all data is labelled.

The performance improvement observed from adding additional unlabelled data may be due to the labelled data being small. The benefit of SSL models is expected to diminish as the size of labelled dataset increases. However, large labelled training datasets are difficult to obtain in clinical applications due to cost constrains and data privacy concerns. The benefit of SSL may be especially useful in training large deep learning models with small datasets. Initializing the end-to-end deep learning model which had 9,218,369 parameters with weights from SSL model improved AUC from 0.73 to 0.8.

We trained a contrastive model such that embeddings of segments from the same patient were closer than embedding from different patients. The pretext task may be well suited for extracting features from PPG and other bio-signals because the underlying physiological parameters are unlikely to change significantly within a short time. The SSL model trained to embed two augmentations of the same PPG segment did not extract useful features despite the pretext task being successful for image tasks, likely due to insufficient data augmentation procedures. Successful augmentation of bio-signals may require introducing noise similar to what is encountered in real life, such as motion artefacts for PPG signals. Audio tasks have benefited from data augmentation techniques such as time stretching and pitch shift, but such augmentation may be unsuitable for bio-signals ( Wei *et al.*, 2020). Time stretching, for instance, would alter the underlying measurement of heart rate, which may negatively affect performance on downstream tasks.

Our study agrees with previous studies that showed that PPG signals are predictive of hospitalization. ( Garde *et al.*, 2016) achieved an AUC of 0.75 in predicting hospitalization using logistic regression based on age, gender, respiratory rate, and hand-crafted features derived from PPG signals using traditional signal processing techniques. Features were extracted from the PPG signal in the time domain based on pulse-to-pulse interval (PPI) statistics, frequency domain after computing the power spectrum density, and additional features computed from SpO _2_. We used SSL for automatic feature extraction eliminating the need for extensive feature engineering or intermediate calculations of SpO _2_. We achieved an AUC of 0.83 using features extracted from PPG without incorporating any clinical features and 0.89 when PPG and clinical features were combined. Although clinical features were more predictive of hospitalization than PPG features (AUC 0.86 vs 0.83), clinical features are subjective and require more effort to collect.

### Implications

The models developed here could be deployed on a mobile phone/tablet with an attached pulse oximeter, and used to augment triage of patients in low income settings. The World Health Organization (WHO) recommends the use of the Emergency Triage and Treatment (ETAT) guidelines for triaging sick children, but challenges remain in its implementation due to staff shortages and adequate training ( King *et al.*, 2021). Therefore, having a model that could objectively identify the need for admission while the triaging nurse is taking measurements of SpO _2_ – which is part of routine clinical practice, could complement ETAT guidelines in identifying severely ill children. Patients identified as severely ill would be fast-tracked to see the facility medical officer/paeditrician as opposed to the first come, first served model that can delay potentially life-saving interventions in busy hospitals. Additional research is required to determine optimal risk cut-offs for triggering action because the implications of false positive and false negatives are not proportional.

Features extracted using self-supervised learning were useful for predicting heart rate, respiratory rate, and SpO _2_ despite the SSL model not being optimized to solve those tasks, implying that the features may contain information on yet unknown physiological parameters contained in the PPG signal. Existence of such unknown parameters could explain the observed improvement in prediction performance of SSL features over models with only SpO _2_ and heart rate (AUC 0.83 vs 0.72), and consequently can enhance the development of novel applications using bio-signals. Such applications may include non-invasive continuous disease monitoring using pulse oximeters, improved triaging of patients seeking care and the development of cheaper and more objective diagnostic tools.

### Limitations

We used SpO _2_ and heart rate measures obtained using a pulse oximeter while the respiratory rate was approximated after tapping on the respiratory rate application with each respiratory cycle. This respiratory rate approximation is a single value (not continuously monitored) and likely to contain noise and may underestimate the predictive performance of extracted features. However, the study does not intend to use machine learning to extract such parameters from PPG waveform for use in clinical setting, but demonstrate that the extracted features contain information about these parameters, and might be useful in predicting outcomes for which the parameters are useful.

Although we used the raw wave-form, the pulse oximeters were from a single manufacturer. Therefore, models trained in this study are unlikely to perform well when applied to devices from different manufacturers without retraining the models. Furthermore, device manufacturers may apply signal pre-processing procedures which are proprietary and implemented in closed source software, making standardization of model development unfeasible. Moreover, it is unclear whether the utility of incorporating unlabelled data using self-supervised learning would remain if the labelled and unlabelled data were collected using pulse oximeters from different manufacturers.

The deep learning models used for both SSL and end-to-end deep learning are computationally intensive compared to traditional signal processing techniques. Consequently, we were not able to estimate the variability in model performance using K-fold cross-validation. However, we used the same test data set to compare all models, and SSL improved performance of all models suggesting the observed benefit is unlikely to be due to chance.

## Conclusion

In conclusion, we demonstrated that SSL using contrastive learning is useful for extracting features from raw PPG signals and initializing end-to-end deep learning models. SSL could speed up the development of novel applications using bio-signals such as PPG by not requiring extensive domain knowledge about the relationship between the waveform and the task of interest. SSL can also leverage large unlabelled data sets to improve the predictive performance of models fitted using small labelled data sets. 

## Data Availability

Harvard Dataverse: Replication Data for: Assessment of Automation of Paediatric Triage using Pulse Oximetry in a Kenyan Public Hospital, https://doi.org/10.7910/DVN/KQ4DNK ( Mwaniki *et al*, 2021). This project contains the following underlying data: Data.csv (Demographic, clinical, and follow-up data of recruited participants) PaediatricTriage_DataDictionary.tab (Data dictionary for Data.csv) pulse_oximeter_data.zip (zipfile containing the red and infrared pulse oximeter waveforms for each patient) The data is under restricted access because it contains potentially identifying information on recruited patients. External data sharing guidelines are available through the KEMRI Data Governance page. Data sharing requests should be addressed to dgc@kemri-wellcome.org. Scholars Portal Dataverse: 6-60m Observation - Pulse Oximetry (dataset) ~ Smart Discharges, https://doi.org/10.5683/SP2/PHX4C5 ( Wiens *et al*, 2021a) Scholars Portal Dataverse: <6m Observation - Pulse Oximetry (dataset) ~ Smart Discharges, https://doi.org/10.5683/SP2/ZDDFZL ( Wiens *et al*, 2021b). The Scholars Portal Dataverse projects are under restricted access. Data requests should be made to the Pediatric Sepsis Data Colab. Analysis code available from: https://github.com/pmwaniki/ppg-analysis/ Archived analysis code as at time of publication: https://doi.org/10.5281/zenodo.7542839 ( Mwaniki, 2023). License: MIT license
