# Supplementary material for: Using self-supervised feature learning to improve the use of pulse oximeter signals to predict paediatric hospitalization
Source: Wellcome Open Res. 2023 Feb 1;6:248. Originally published 2021 Sep 28. [Version 2] doi: 10.12688/wellcomeopenres.17148.2 (PMC10280037; doi:10.12688/wellcomeopenres.17148.2)
Supplement: Peer review history

# Peer review: Reviewer response for version 2

**DOI:** 10.21956/wellcomeopenres.20943.r54625

**Published:** 2023-06-15

**Authors:** Ciira wa Maina

**Affiliations:** 1 Department of Electrical and Electronics Engineering, Dedan Kimathi University of Technology, Nyeri, Kenya

I have reviewed the resubmission and note the changes made based on the initial review.

 The discussion of the tSNE visualisation is still unclear as tSNE it is usually informative when clusters are clearly visible. Efforts should be made to clearly explain how we should interpret the tSNE plots. Alternatively, the tSNE visualisations can be eliminated without affecting the manuscript in my opinion.

 Minor comments

1. In the discussion edit the word "constrains" to "constraints".

Is the work clearly and accurately presented and does it cite the current literature?

Partly

If applicable, is the statistical analysis and its interpretation appropriate?

Partly

Are all the source data underlying the results available to ensure full reproducibility?

Yes

Is the study design appropriate and is the work technically sound?

Yes

Are the conclusions drawn adequately supported by the results?

Yes

Are sufficient details of methods and analysis provided to allow replication by others?

Yes

Reviewer Expertise:

Machine learning, digital signal processing

I confirm that I have read this submission and believe that I have an appropriate level of expertise to confirm that it is of an acceptable scientific standard.

# Peer review: Reviewer response for version 2

**DOI:** 10.21956/wellcomeopenres.20943.r54626

**Published:** 2023-04-11

**Authors:** Shaun Davidson

**Affiliations:** 1 Department of Engineering Science, Institute of Biomedical Engineering, University of Oxford, Oxford, UK

This reviewer feels that the changes made by the authors, in particular the comparison between self-supervised learning models and models using clinical features as inputs, as well as the explanation of the potential clinical impact of the work, have significantly improved the paper.

Is the work clearly and accurately presented and does it cite the current literature?

Partly

If applicable, is the statistical analysis and its interpretation appropriate?

Partly

Are all the source data underlying the results available to ensure full reproducibility?

Yes

Is the study design appropriate and is the work technically sound?

Yes

Are the conclusions drawn adequately supported by the results?

Partly

Are sufficient details of methods and analysis provided to allow replication by others?

Yes

Reviewer Expertise:

Circadian rhythms, wearable technology, non-contact vital sign monitoring

I confirm that I have read this submission and believe that I have an appropriate level of expertise to confirm that it is of an acceptable scientific standard.

# Peer review: Reviewer response for version 1

**DOI:** 10.21956/wellcomeopenres.18941.r49279

**Published:** 2022-07-25

**Authors:** Shaun Davidson

**Affiliations:** 1 Department of Engineering Science, Institute of Biomedical Engineering, University of Oxford, Oxford, UK

This paper explores using self-supervised learning to automatically detect PPG features best suited to predicting paediatric hospitalisation from a set of 60-second recordings. These features are then fed into both a logistic regression and deep learning model, and performance is assessed. The features derived via self-supervised learning were found to improve performance compared to random model initialisation. The paper is well-written and methodologically interesting. Additionally, the authors are to be commended for making the Python code of their methodology available to readers. However, this reviewer feels that there are several improvements to the manuscript that should be made.

1. The authors note that " *self-supervised models performed better when trained on labelled and unlabelled data...*" (Discussion, paragraph 1). This reviewer feels that this statement is too broad and needs to be framed in the context of the relatively small size of the labelled data set (1031 recordings) and the larger size of the unlabelled dataset (approx. 13000 recordings). Clearly, additional unlabelled data can improve self-supervised learning in cases where only a small labelled dataset is available, and, as such cases are common, this result has clear and important benefits. However, the statement as written could be read as suggesting that adding unlabelled data, regardless of the size of your labelled or unlabelled dataset, improves the results of self-supervised learning, which this reviewer does not feel is supported by the results in the paper and is unlikely to be the case (a sufficiently large labelled dataset would likely perform identically or better, but require significant more manual annotation).
2. This reviewer feels (in line with reviewer 1) that the comparison between a model initialised using self-supervised learning compared to random initialisation is not quite a 'like-for-like' comparison. Most PPG algorithms, as stated in the manuscript's introduction, tend to use hand-crafted features derived from decades of signal processing and medical work. Surely it would be worth comparing the results of the self-supervised learning methods with these methods which are more representative of the existing 'state-of-the-art' and involve an equivalent, if different and manual, signal pre-processing layer.
3. This reviewer feels that the novelty and clinical relevance of this work need to be made more clear. While self-supervised learning can be useful in cases where " *domain knowledge on the relationship between the waveform and the task of interes*" is not available, PPG is definitely not one of these cases (as mentioned in the previous point). Additionally, despite all of this existing domain knowledge and the general availability of PPG signals, this reviewer is not aware of any clinical desire or inclination to move towards using PPG as a sole means of triage (as opposed to using PPG-derived HR and SpO2 as part of a broader framework of vital signs for triage). How would the authors expect this approach to be received by clinicians or employed in the clinic, and what benefits do they feel it could provide?

Is the work clearly and accurately presented and does it cite the current literature?

Partly

If applicable, is the statistical analysis and its interpretation appropriate?

Partly

Are all the source data underlying the results available to ensure full reproducibility?

Yes

Is the study design appropriate and is the work technically sound?

Yes

Are the conclusions drawn adequately supported by the results?

Partly

Are sufficient details of methods and analysis provided to allow replication by others?

Yes

Reviewer Expertise:

Circadian rhythms, wearable technology, non-contact vital sign monitoring

I confirm that I have read this submission and believe that I have an appropriate level of expertise to confirm that it is of an acceptable scientific standard, however I have significant reservations, as outlined above.

# Peer review: Reviewer response for version 1

**DOI:** 10.21956/wellcomeopenres.18941.r46193

**Published:** 2021-11-02

**Authors:** Ciira wa Maina

**Affiliations:** 1 Department of Electrical and Electronics Engineering, Dedan Kimathi University of Technology, Nyeri, Kenya

The paper presents a model to predict paediatric hospitalisation using the raw photoplethysmography signal obtained from a pulse oximeter. This model relies on self supervised learning to initialise deep neural networks to overcome the difficulty of training deep learning models from scratch using limited data.

**Comments**

1. The justification for using the ResNet 50 model for the encoder is not clear. Given the limited data, it is possible that a simpler model could lead to better performance. 
2. The tSNE visualisations are confusing. What clustering is expected here? Perhaps moving to 3D would improve the visualisation.
3. This paper should compare the predictions based on the neural network with a model that extracts parameters of interest such as heart rate, respiratory rate and oxygen saturation from the raw photoplethysmography signal and then uses these to predict paediatric hospitalisation.
4. The authors should comment on how this model would be deployed and used in the clinic.
5. In the comparison between the predicted values of heart rate and the labelled values (Figure 2) the authors should compare the values obtained to other methods in the literature. In addition, what influence does the anatomical location from which the signal is obtained have on the accuracy?

**Minor Comments**

1. In describing the Gaussian noise used to corrupt the signal, signal-to-noise ratio is more useful that the variance in absolute terms.
2. In the description of the terms in Equation 1, the ‘i’ in ‘ki’ should be a subscript.
3. On the last paragraph of page 7, edit the line “However, we used the save test data set to compare…”
4. In the conclusion, include a space in “... models.SSL...”

Is the work clearly and accurately presented and does it cite the current literature?

Partly

If applicable, is the statistical analysis and its interpretation appropriate?

Partly

Are all the source data underlying the results available to ensure full reproducibility?

Yes

Is the study design appropriate and is the work technically sound?

Yes

Are the conclusions drawn adequately supported by the results?

Yes

Are sufficient details of methods and analysis provided to allow replication by others?

Yes

Reviewer Expertise:

Machine learning, digital signal processing

I confirm that I have read this submission and believe that I have an appropriate level of expertise to confirm that it is of an acceptable scientific standard, however I have significant reservations, as outlined above.
